# The impact of library preparation protocols on the consistency of allele frequency estimates in Pool‐Seq data

**DOI:** 10.1111/1755-0998.12432

**Published:** 2015-06-09

**Authors:** Robert Kofler, Viola Nolte, Christian Schlötterer

**Affiliations:** ^1^ Institut für PopulationsgenetikVetmeduni ViennaVeterinärplatz 1, 1210 WienAustria

**Keywords:** Drosophila, NGS libraries, Pool‐Seq, population genetics—empirical

## Abstract

Sequencing pools of individuals (Pool‐Seq) is a cost‐effective method to determine genome‐wide allele frequency estimates. Given the importance of meta‐analyses combining data sets, we determined the influence of different genomic library preparation protocols on the consistency of allele frequency estimates. We found that typically no more than 1% of the variation in allele frequency estimates could be attributed to differences in library preparation. Also read length had only a minor effect on the consistency of allele frequency estimates. By far, the most pronounced influence could be attributed to sequence coverage. Increasing the coverage from 30‐ to 50‐fold improved the consistency of allele frequency estimates by at least 27%. We conclude that Pool‐Seq data can be easily combined across different library preparation methods, but sufficient sequence coverage is key to reliable results.

## Introduction

The dramatic reduction in sequencing costs since the advent of the next‐generation sequencing technology has changed biology by allowing to tackle many new research questions that could not be addressed before. Despite this success, the analysis of natural populations on a genomic scale remained still prohibitively expensive. With allele frequency estimates being key to population genetic analysis, Pool‐Seq provides an affordable approach to study population variation on a genomic scale (Schlötterer *et al*. 2014). Short sequence reads obtained from a large pool of individuals sample the allelic variation across the entire genome. The major cost advantage compared to sequencing of individuals separately stems from the fact that each sequence read contributes to the accuracy of the allele frequency estimate. In contrast, sequencing of individuals typically uses several reads for each allele, which makes it less cost‐effective than Pool‐Seq (Futschik & Schlötterer 2010; Gautier *et al*. 2013; Schlötterer *et al*. 2014).

Due to the cost‐effectiveness of Pool‐Seq, this method has been applied to a broad range of research questions, such as the identification of the genetic basis of complex traits (Bastide *et al*. 2013), mapping of genes involved in domestication (Rubin *et al*. 2010), tracking of selected alleles in evolving populations (Orozco‐terWengel *et al*. 2012), monitoring the evolution of cancer cells during treatment (Ding *et al*. 2012) and the invasion of transposable elements (Kofler *et al*. 2015).

This versatility of Pool‐Seq in combination with a growing number of software tools specifically designed for the analysis of Pool‐Seq data (Schlötterer *et al*. 2014) results in a steadily growing number of publically available Pool‐Seq data (e.g. at the European Nucleotide Archvie: http://www.ebi.ac.uk/ena). One particularly good example is *Drosophila melanogaster*, a species for which natural populations from different continents have been analysed separately (Kolaczkowski *et al*. 2011; Fabian *et al*. 2012; Bastide *et al*. 2013; Tobler *et al*. 2013; Bergland *et al*. 2014). It is apparent that the joint analysis of these data sets provides an enormous potential to understand key parameters of the biology of *D. melanogaster*. However, as the publicly available Pool‐Seq data are typically contributed by different groups using a diverse set of library preparation protocols or bioinformatics pipelines, it is not clear whether these data yield consistent estimates of allele frequencies, a requirement for performing an unbiased meta‐analysis.

While it has been documented previously that consistent bioinformatic procedures are key to reliable allele frequency estimates (Kofler *et al*. 2011a), the influence of the library preparation protocol has not yet been investigated.

Aiming to fill this gap, we evaluated the consistency of allele frequency estimates from Pool‐Seq data using four different library protocols applied to the same DNA extracted from *D. melanogaster* and *Drosophila simulans* individuals. We show that despite some influence of the library preparation protocol, this effect is minor compared to the error introduced by low sequence coverage. Hence, with consistent and adequate bioinformatic processing, it is possible to perform meta‐analyses of different Pool‐Seq data sets.

## Material and methods

### DNA extraction and library preparation

We extracted genomic DNA from 554 *Drosophila melanogaster* and 793 *Drosophila simulans* individuals using standard DNA extraction methods, previously applied to Pool‐Seq experiments (Tobler *et al*. 2013; Franssen *et al*. 2015). Four different library preparation protocols were used. Two of them involved a PCR amplification step: the NEBNext Ultra (+) protocol was based on the NEBNext^®^ Ultra DNA Library Prep Kit (E7370L) and the NEBNext DNA (+) protocol used the NEBNext^®^ DNA Library Prep Master Mix Set (E6040L). The other two were PCR‐free protocols: the NEXTflex (−) protocol based on the NEXTflex^™^ PCR‐Free DNA Sequencing Kit (5142‐02) and the TruSeq (−) protocol using the TruSeq DNA PCR‐Free Sample Preparation Kit (FC‐121‐3001).

All libraries were produced using slight modifications of the standard protocols except for the TruSeq (−) libraries, which were prepared according to the supplier's instructions.

The following amount of genomic DNA was used: 1 μg for the NEBNext Ultra (+), 3 μg for the NEXTflex (−), 1 μg for the NEBNext DNA (+) and 2 μg for the TruSeq (−) protocol. For all libraries, genomic DNA was fragmented using a Covaris S2 device (Covaris Inc., Woburn, MA, USA) with the following settings: 3 × 20 s at 10% duty cycle, intensity 5 and 200 cycles per burst.

End repair, A‐tailing and ligation were performed according to the suppliers' protocols except that the adapters for the NEBNext DNA (+) libraries were taken from the TruSeq DNA LT Sample Prep Kit (FC‐121‐2001). Identical barcodes were chosen for each sample across protocols. Purification steps within the NEBNext DNA (+) protocol were performed using Qiagen columns (Qiagen, Hilden, Germany).

An initial size selection was performed using AMPureXP beads (Beckman Coulter, CA, USA), either before the PCR step (for the two PCR‐based protocols), or at the end of the protocol (for the NEXTflex (−) protocol).

The NEBNext Ultra (+) samples were amplified using Phusion Polymerase included in the NEBNext Ultra DNA Kit, and the NEBNext master mix samples using the master mix included into the TruSeqDNA LT Sample Prep Kit. Both PCR‐based protocols used the following cycling conditions: an initial denaturation step at 98 °C/30 s, followed by 10 cycles at 98 °C/10 s, 65 °C (Phusion polymerase) or 60 °C (TruSeq polymerase)/30 s, 72 °C/50 s and a final extension at 72 °C/7 min.

Libraries made with the two PCR‐based and the NEXTflex (−) protocol were further size‐selected on an agarose gel to yield a narrow total fragment size range of 400–430 bp. For the two PCR‐based sets of libraries, size selection was carried out before the PCR step, for the NEXTflex (−)‐based libraries at the end of the protocol. The narrow size range has been used to facilitate the analysis of TE insertions (Kofler *et al*. 2012). The bead‐based size selection for the TruSeq (−) libraries followed the instructions for 350 bp insert sizes in the manual without an additional gel‐based size selection. Sequencing libraries were prepared separately for each combination of species (2), replicate (2) and protocol (4) (4 × 2 × 2 = 16), and a total of six lanes 2 × 100 bp paired‐end reads were sequenced on a HiSeq2000 (Illumina, San Diego, CA, USA). Three lanes were run for NEXTflex (−) protocol and one lane for each of the other protocols.

### Bioinformatic analyses

2 × 100 bp reads were trimmed with PoPoolation (r226) (Kofler *et al*. 2011a) and mapped on a Hadoop cluster using the DistMap tool (Pandey & Schlötterer 2013), which implements bwa (v0.7.5a) (Li & Durbin 2010), to the reference genome of *D. melanogaster* (v5.53) or of *D. simulans* (v1.0) (Palmieri *et al*. 2015).

The trimming and mapping parameters as well as the masking of repetitive regions followed previous Pool‐Seq experiments (Tobler *et al*. 2013; Franssen *et al*. 2015). Mapping statistics were obtained with Picard (http://broadinstitute.github.io/picard) and custom Python scripts. We generated mpileup files with samtools (v0.1.18) (Li *et al*. 2009) and sync files with PoPoolation2 (r196) (Kofler *et al*. 2011b) using a minimum quality of 20. Only the major chromosomes (X, 2L, 2R, 3L, 3R, 4) were analysed, and regions around indels (window of 5 bp) were filtered. Coverage (i.e. average number of reads covering a given position in the genome) was standardized with PoPoolation2 (Kofler *et al*. 2011b).

A set of high‐quality SNPs was called across all libraries using a minimum sequence quality of 40 and a minimum count of three (*D. melanogaster*: 3 983 099 SNPs, *D. simulans*: 5 187 418 SNPs). Allele frequency differences were only calculated for high‐quality SNPs with the required minimum coverage in all libraries.

Reads were trimmed at the 3′ end to generate reads of size 50, and the whole protocol, including the mapping of the reads, was repeated.

We quantified the error introduced by the library preparation as:

$$E=\frac{d_{\mathrm{between}}-d_{\mathrm{within}}}{d_{\mathrm{between}}}\times 100$$

with *d* being the absolute allele frequency difference across all SNPs. *d*_within_ is the average of the technical replicates, and *d*_between_ is the average of all possible comparisons between different library preparations.

## Results

We extracted DNA from a large pool of *Drosophila melanogaster* and *Drosophila simulans* individuals to generate the starting material for four different library preparation protocols, which were each performed in duplicate. We produced 1.65×10^9^ paired‐end 100 bp sequence reads, which corresponds to an average of 1×10^8^ reads per library. With the libraries from each species being generated from the same starting material, unbiased libraries are expected to result in the same allele frequency estimate from the Pool‐Seq data.

As expected for the better quality of the *D. melanogaster* genome, we observed a higher fraction of mapped reads, fewer broken pairs and a smaller variation in coverage compared with *D. simulans* (Table 1). Interestingly, all mapping statistics were highly similar among replicates of the same library preparation protocol. When averaged over species and replicates, we found that the PCR‐free NEXT‐flex protocol had the lowest fraction of mapped reads and the highest coverage variation. As expected, the two PCR‐based library protocols had the highest fraction of PCR duplicates, but also for PCR‐free libraries, we detected a considerable fraction of duplicates. The fraction of chimera was higher in the PCR‐free libraries, a phenomenon, which had already been noted before (Oyola *et al*. 2012). Importantly, these quality indicators were more variable between species than among library preparation protocols, suggesting that the choice of library preparation protocol is of less importance than the quality of the reference genome.

**Table 1:** Mapping statistics for Pool‐Seq data generated with different library preparation protocols from genomic DNA of *Drosophila melanogaster* (Dmel) and *Drosophila simulans* (Dsim); data are shown for two PCR‐free protocols (−) and two protocols using PCR amplification (+); Rep.: replicates; Reads: reads in million; m.: mapped reads in percent; br.p.: broken pairs, that is paired‐end fragments not mapped as proper pair, in percent; Error: sequencing error in percent (including polymorphism); Indel: indel error in percent (including polymorphism); Chi.: chimera, that is paired‐end fragments where reads map to discordant positions, in percent; Dup.: duplicates in percent; Cov. CV: coefficient of variation for the coverage

	Protocol	Rep.	Reads	m. (%)	br.p (%)	Error (%)	Indel (%)	Chi. (%)	Dup.^a^(%)	Cov. CV^a^
Dmel	NEBNext Ultra (+)	1	79	96.3	2.0	0.76	0.055	0.88	4.03	0.29
		2	103	96.3	2.0	0.77	0.055	0.94	4.00	0.29
	NEXTflex (−)	1	162	95.0	4.7	0.65	0.046	3.04	2.46	0.40
		2	199	94.2	4.2	0.65	0.047	2.5	2.07	0.34
	NEBNext DNA (+)	1	90	96.2	2.3	0.73	0.054	1.03	3.76	0.25
		2	96	96.1	2.1	0.73	0.053	0.92	3.22	0.24
	TruSeq (−)	1	76	96.8	2.4	0.76	0.056	1.22	2.15	0.27
		2	84	96.7	3.0	0.76	0.056	1.74	1.95	0.27
Dsim	NEBNext Ultra (+)	1	74	85.6	2.1	1.36	0.101	0.76	2.64	0.41
		2	64	85.6	2.1	1.36	0.102	0.77	2.94	0.42
	NEXTflex (−)	1	133	87.5	5.6	1.21	0.086	3.26	1.49	0.53
		2	137	87.4	5.0	1.25	0.094	2.82	2.54	0.45
	NEBNext DNA (+)	1	79	85.3	2.5	1.32	0.100	0.93	2.79	0.41
		2	90	85.9	2.2	1.32	0.100	0.8	2.22	0.41
	TruSeq (−)	1	92	86.5	2.6	1.37	0.104	0.93	0.68	0.37
		2	90	86.5	2.9	1.37	0.103	1.19	0.68	0.37

a For statistics that are sensitive to coverage differences we subsampled the data to 26 930 986 proper pairs across all sample.

To evaluate the consistency of the allele frequency estimates across libraries, we downsampled the reads to a homogeneous coverage across the entire genome. The consistency of the allele frequency estimates *d* was determined as the average difference in allele frequency, either between all pairs of libraries produced with the same protocol (*d*_within_ *)* or all possible combinations of pairs involving two different library preparation protocols (*d*_between_). In agreement with previous results (e.g. Kofler *et al*. 2011a), we found that coverage was the primary determinant of the consistency of the allele frequency estimate (Table 2). Increasing the coverage from 30 to 50 resulted in about a 27% more consistent allele frequency estimate. Decreasing the read length from 100 to 50 bp paired‐end reads increased the inaccuracy by 0.8–1.1%. The difference in allele frequency estimate introduced by the library preparation protocol (*E*) ranged from 0.4% to 0.8%, which corresponds to about 10% of the total difference in allele frequency estimate.

**Table 2:** Average allele frequency difference within (*d*_within_) and between (*d*_between_) the library preparation protocols, for different coverages (cov.) in data from *Drosophila melanogaster* (Dmel) and *Drosophila simulans* (Dsim); *E*: error due to library preparation (%); snps: number of SNPs analysed

	Cov.	SNP	*d*_within_	*d*_between_	E
Dmel	30	2 977 317	0.0548	0.0551	0.5558
	40	1 954 383	0.0466	0.0468	0.578
	50	592 354	0.04	0.0402	0.5384
Dsim	30	2 992 813	0.0597	0.06	0.5153
	40	1 032 206	0.0502	0.0504	0.4042
	50	119 047	0.0427	0.0431	0.7862

**Table 3:** Assignment of accession nos to sequencing libraries

	Protocol	r1	r2
Dmel	NEBNext Ultra (+)	ERR557048	ERR557049
	NEXTflex (−)	ERR557050, ERR557052, ERR557054	ERR557051, ERR557053, ERR557055
	NEBNext DNA (+)	ERR557056	ERR557057
	TruSeq (−)	ERR832532	ERR832533
Dsim	NEBNext Ultra (+)	ERR557058	ERR557059
	NEXTflex (−)	ERR557060, ERR557062, ERR557064	ERR557061, ERR557063, ERR557065
	NEBNext DNA (+)	ERR557066	ERR557067
	TruSeq (−)	ERR832534	ERR832535

Dmel, *Drosophila melanogaster*; Dsim, *Drosophila simulans*; r1, replicate 1; r2, replicate 2.

## Discussion

The goal of this study was the comparison of different NGS library preparation protocols, with a special emphasis on the Pool‐Seq application. As we used DNA from a large pool of individuals, it was not feasible to determine the genotypes of each individual contributing to the pool separately. Thus, the true allele frequencies were not known. In our analysis, we did not compare the deviation from the true allele frequency, but the differences between two libraries generated from the same source DNA. Hence, we estimated the consistency of allele frequency estimates rather than the accuracy. However, in the absence of systematic errors of Pool‐Seq, the consistency will reflect the accuracy of allele frequency estimate obtained with Pool‐Seq. As several studies have validated Pool‐Seq as an unbiased approach to determine allele frequencies (e.g. Rellstab *et al*. 2013; Zhu *et al*. 2012), such systematic errors are probably rare and our consistency estimates thus likely reflect the accuracy of Pool‐Seq.

Using a broad range of mapping quality estimators, such as percent mapped reads, percent broken pairs and sequence homogeneity, we detected some minor differences between the library preparation protocols evaluated. Strikingly, a much more pronounced effect was seen for these estimators when the *Drosophila melanogaster* and *Drosophila simulans* reference genomes were contrasted. The quality of these two reference genomes differs markedly. While the *D. melanogaster* genome is one of the best reference genomes available, the *D. simulans* genome was assembled from short read sequence data only. Many of the mapping quality estimators in Table 1 clearly reflect these quality differences in the reference genomes.

Interestingly, we also noticed a lower consistency among libraries prepared from *D. simulans* DNA. Assemblies from short read data are more likely to suffer from collapsed regions of high similarity (e.g. gene families) and missing repetitive sequences (e.g.: transposable elements). It is well documented that this could result in the erroneous identification of SNPs (e.g. Phillippy *et al*. 2008), but it is not apparent why this would cause a lower consistency in allele frequency estimates between library preparations. Rather, we attribute the difference between libraries for the two species to different levels of polymorphism. Irrespective of sequence coverage, we find that *D. simulans* has a higher SNP heterozygosity than *D. melanogaster* (coverage 30 Dmel = 0.201, Dsim = 0.216; coverage 40 Dmel = 0.194, Dsim = 0.206; coverage 50 Dmel = 0.182, Dsim = 0.193). As the binominal sampling error increases up to an allele frequency of 0.5, a higher sampling error is expected for SNPs with higher heterozygosity. We conclude that the different levels of variability between the two species are a more likely explanation for the variation in consistency of allele frequency estimates between the two species than the quality of the reference genomes.

In addition to the differences between the two species, we also noticed a systematic effect of the library preparation protocol on the consistency of allele frequency estimates. Nevertheless, this error was only about 0.5%, which corresponds to 10% of the error between technical replicates. In the light of these results, we conclude that library preparation protocols do not strongly affect Pool‐Seq results. This opens the possibility of meta‐analyses, combining results obtained with different library preparation protocols. We caution, however, that reproducible results will be highly contingent on using a comparable mapping pipeline, as mapping parameters have been previously shown to affect allele frequency estimates in Pool‐Seq data (Kofler *et al*. 2011a). Thus, we recommend remapping of reads with the same pipeline to make data suitable for meta‐analysis. Importantly, this strategy has already been used for *D. melanogaster* data, which were processed according to a standardized bioinformatics protocol to facilitate meta‐analyses (Lack *et al*. 2015).

Our conclusion that the differences between library preparation protocols are so small relative to other sources of variation may be specific to the Pool‐Seq application. Another study comparing different library preparation protocols identified clear differences and recommended to select the library preparation protocol according to the goals of the study (Rhodes *et al*. 2014).

C.S. conceived the experiment, V.N. generated the data, R.K. analysed the data, R.K., V.N., C.S. wrote the manuscript.

## Data Accessibility

The data are publicly available from the European Nucleotide Archive (http://www.ebi.ac.uk/ena Table 3). All scripts, the set of high‐quality SNPs and the sync files (containing allele counts for all samples) are available from Dryad (http://datadryad.org/ doi:10.5061/dryad.p31j8).
